# Development and validation of prognostic models for colon adenocarcinoma based on combined immune-and metabolism-related genes

**DOI:** 10.3389/fonc.2022.1025397

**Published:** 2022-10-31

**Authors:** Hui-zhong Jiang, Bing Yang, Ya-li Jiang, Xun Liu, Da-lin Chen, Feng-xi Long, Zhu Yang, Dong-xin Tang

**Affiliations:** ^1^ College of Graduate, Guizhou University of Traditional Chinese Medicine, Guiyang, China; ^2^ The First Affiliated Hospital of Guizhou University of Traditional Chinese Medicine, Guiyang, China

**Keywords:** colon adenocarcinoma, immune, metabolism, prognosis, tumor microenvironment

## Abstract

**Background:**

The heterogeneity of tumor tissue is one of the reasons for the poor effect of tumor treatment, which is mainly affected by the tumor immune microenvironment and metabolic reprogramming. But more research is needed to find out how the tumor microenvironment (TME) and metabolic features of colon adenocarcinoma (COAD) are related.

**Methods:**

We obtained the transcriptomic and clinical data information of COAD patients from The Cancer Genome Atlas (TCGA) and Gene Expression Omnibus (GEO) databases. Consensus clustering analysis was used to identify different molecular subtypes, identify differentially expressed genes (DEGs) associated with immune-and metabolism-related genes (IMRGs) prognosis. Univariate and multivariable Cox regression analysis and Lasso regression analysis were applied to construct the prognostic models based on the IMRG risk score. The correlations between risk scores and TME, immune cell infiltration, and immune checkpoint genes were investigated. Lastly, potential appropriate drugs related to the risk score were screened by drug sensitivity analysis.

**Results:**

By consensus clustering analysis, we identified two distinct molecular subtypes. It was also found that the multilayered IMRG subtypes were associated with the patient’s clinicopathological characteristics, prognosis, and TME cell infiltration characteristics. Meanwhile, a prognostic model based on the risk score of IMRGs was constructed and its predictive power was verified internally and externally. Clinicopathological analysis and nomogram give it better clinical guidance. The IMRG risk score plays a key role in immune microenvironment infiltration. Patients in the high-risk groups of microsatellite instability (MSI) and tumor mutational burden (TMB) were found to, although with poor prognosis, actively respond to immunotherapy. Furthermore, IMRG risk scores were significantly associated with immune checkpoint gene expression. The potential drug sensitivity study helps come up with and choose a chemotherapy treatment plan.

**Conclusion:**

Our comprehensive analysis of IMRG signatures revealed a broad range of regulatory mechanisms affecting the tumor immune microenvironment (TIME), immune landscape, clinicopathological features, and prognosis. And to explore the potential drugs for immunotherapy. It will help to better understand the molecular mechanisms of COAD and provide new directions for disease treatment.

## Introduction

Colon adenocarcinoma (COAD) is a leading cause of cancer-related mortality and one of the most frequent cancers worldwide. New treatment strategies are desperately needed to address the rising global patient population. Immunotherapy has become a pivotal role in cancer treatment programs, especially the immune checkpoint inhibitor (ICI) therapy, which has become the most promising treatment method. Mismatch repair deficiency (dMMR) and high microsatellite instability (MSI-H) tumors are now treated first with ICI therapy for COAD. However, there is no efficacy in COAD with mismatch repair proficiency (pMMR) and low microsatellite instability (MSI-L) or microsatellite stability (MSS) (1). Traditional chemotherapy is still the gold standard for this patient subset. It’s possible that the heterogeneity of solid tumors and their surrounding microenvironment are to blame for this finding (2).

Tumor metabolism is a well-recognized feature of cancer (3). For cancer cells to rapidly proliferate, metabolic reprogramming is crucial because it provides the cells with the energy they need to multiply. Meanwhile, the tumor immune microenvironment (TIME) is well-nourished, allowing cancer cells to thrive (4). Thus, tumor cells provide a good metabolic environment for themselves. When tumor cells secrete metabolites, they can have an effect on immune cells and alter the TIME. Meanwhile, tumor cells and immune cells competing for energy demands can block T cell activation and proliferation. Tumor cells express immune checkpoint proteins PD-1 and CTLA-4, which inhibit T cell metabolism (5). Research shows the specific metabolism of immune cells can also lead to tumor cells developing immune escape (6). Eventually, the immune escape of tumor cells will affect the clinical treatment effect. This implies the immune system is the umbrella of the body, while immune escape is a safe house for tumors. Because of how complicated the relationship is between metabolism and immunity, it is especially important to construct and validate prognostic models that combine immune and metabolic features of COAD patients to help with immunotherapy. Currently, prognostic models have been constructed for single immune or single metabolic related genes. The prognostic model constructed with 11 metabolism-related genes can be used to predict treatment response and to define the biomarkers of metabolic therapy in COAD patients (7). Furthermore, the development of a prognostic model based on 18 immune-related genes could indicate immune cell infiltration and demonstrate their critical role in TIME (8). Some studies consider immune score and consensus molecular subtype classification as promising biomarkers for predicting the efficacy of selected COAD treatments (9). For immunotherapy in COAD, more biomarkers will also need to be mined to understand the molecular mechanisms controlling immune-and metabolism-related genes (IMRGs) and to predict their relationship to therapy (10). These will provide new perspectives and more personalized treatment options for targeted oncology options.

In this study, we combined multiple datasets to develop and validate a novel prognostic model based on IMRGs. Meanwhile, we comprehensively explored the association of this feature to the immune landscape, immunotherapy response, and drug sensitivity of COAD patients. Our results demonstrate that our constructed features based on IMRGs can be used as potential biomarkers to predict the clinical outcome and immunotherapy efficacy in COAD patients.

## Marerials and methods

### Data collection and preprocessing

The Cancer Genome Atlas database (https://portal.gdc.cancer.gov/, TCGA) was used to find the transcriptomic data (fragments per kilobase million, FPKM), clinical data, and somatic mutation data of COAD patients. A total of 521 TCGA-COAD samples were obtained, including 41 normal samples and 480 COAD tumor samples. Preprocessing converted the FPKM values of the TCGA-COAD to the transcripts per million (TPM). The Gene Expression Omnibus database (https://www.ncbi.nlm.nih.gov/geo/, GEO) obtained samples containing survival outcome information, and the GSE40967 cohort (11) and GSE17536 cohort (12) contained 585 samples and 177 samples, respectively. Gene expression data from the three datasets were merged and batch corrected by the “ComBat” algorithm in the R package “sva”, leaving a total of 1211 samples for subsequent analysis. 2,483 immune-related genes were obtained from the ImmPort database (13) (https://www.immport.org). By downloading the “c2.cp.kegg.v7.4.symbols” from the MSigDB, we extracted 816 metabolism-related genes. After combining immune-related genes with metabolism-related genes and getting rid of duplicates, a total of 2597 IMRGs were left to study further.

### Consensus cluster analysis of IMRGs

Differentially expressed genes (DEGs) of IMRGs in COAD tumor samples and normal samples were analyzed by the “limma” package. |logFC| > 1 and FDR < 0.05 were set as the criterion for screening DEGs, and differentially expressed IMRGs were extracted. The “ggplot2” package draws volcano maps, the Gene Ontology (GO), the Kyoto Encyclopedia of Genes and Genomes (KEGG), and the Disease Ontology (DO) analysis to identify enriched GO terms, associated signaling pathways, and diseases. Consensus clustering analysis of the extracted DEGs using the “ConsesusClusterPlus” package (14) divided the clusters of different IMRGs characterized. And principal component analysis (PCA) was performed to distinguish the clusters of IMRGs. After extracting survival information from clinical data and removing data with survival times less than 30 days, the overall survival (OS) contrasts between clusters of different IMRGs were compared by Kaplan-Meier analysis. Heatmap visualizing the relationship between clinical pathological features and clusters of different IMRGs. The gene set variation analysis (GSVA) to compare the biological functional differences between the clusters of different IMRGs by the “GSVA” package (15). In the “GSVA” and “GSVABase” packages, a single-sample gene enrichment analysis of the 23 immune cell-related gene sets (ssGSEA) (16) was performed to assess the relative abundance of immune cell infiltration between the different IMRG clusters.

### Construction of a prognostic model for IMRGs

A univariate Cox regression analysis was performed on the DEGs in this study to identify the genes associated with the COAD prognosis. Unsupervised clustering based on prognostic IMRG expression will also be used to classify patients into different subtype groups, namely, gene subtype A and gene subtype B. All COAD patients were randomized into the training group (n = 577) and the test group (n = 578), and the IMRGs risk score with prognosis was constructed, combining with the previous results. A risk prediction model was established by performing the Lasso Cox regression algorithm using the “caret”, and “glmnet” packages. Candidate genes were selected using multivariate Cox analysis to establish a prognostic IMRGs risk score in the training set. The calculation formula is as follows: risk score =Σ(EXPI×coefi), coefi while EXPI represents the respective risk coefficient and expression level of each gene. The total sample, training group, and test group were each divided into high-risk and low-risk groups according to the median risk score. The Kaplan-Meier survival analysis and the receiver operating characteristics (ROC) were generated.

### Correlation between prognostic IMRGs risk scores and clinical subtypes

Sankey plots show patients’ relationships between IMRG clusters, gene clusters, IMRG risk scores, and survival status. WilcoxTest compares the difference in IMRGs risk scores between different IMRGs clusters or prognostic gene clusters. The relationship between the IMRGs risk score and the clinical characteristics (age, gender, clinical stage, and TNM) was further explored. Meanwhile, we performed univariate and multivariate Cox analyses on training and test sets to judge the independent prognostic role of IMRGs risk score. We did stratified analyses to see if the risk scores from IMRGs still worked as good predictors in different age, gender, clinical stage, and TNM subgroups.

### Build a nomogram and validation

Using the “rms” program, we created prediction nomograms based on the independent prognostic analysis, which included clinical features and IMRGs risk ratings (17). The nomograms were analyzed using ROC curves that varied over time to account for 1-, 3-, and 5-year survival rates. Nomogram calibration plots displayed the concordance between observed and anticipated 1-, 3-, and 5-year survival rates. To get a better idea of the nomogram’s predictive power, we ran a decision curve analysis (DCA) using the “ggDCA” package.

### Immunolandscape analysis

Multiple methods were compared in order to conduct a thorough examination of immune infiltration and function. The “ESTIMATE” system (18) can score the immune cell content and matrix content to forecast the immune infiltration and matrix condition of the tumour microenvironment (TME). The CIBERSORT algorithm (19) was used to visualize the proportion of 22 immune-related cell subtypes in different groups, and the “tidyverse” and “ggExtra” packages recycled all immune cells to obtain the correlation between the risk score and immune cells. The putative immunomodulatory processes are analyzed by scoring the immune function and immune cells in the high-risk and low-risk groups using single-sample gene enrichment analysis (ssGSEA). The TIMER2.0 database (http://timer.cistrome.org) downloaded the immune cell infiltration estimation file of TCGA (20), evaluated the immune infiltration and function of the high-risk and low-risk groups, including TIMER (21), CIBERSORT (22), quanTIseq (23), xCell (24), MCP-counter (25), and EPIC (26), drawing the heatmap to centrally display the results of the analysis. Also, we analysed the 47 immune checkpoint genes across the high-risk and low-risk groups, looking for commonalities and discrepancies. The TCIA database (https://tcia.at/home) was used to get the Immunoapparent score (IPS) for COAD patients. This score was used to measure how well high-risk and low-risk groups responded to immunotherapy.

### Correlation of the risk scores of IMRGs with tumor mutation burden (TMB) and MSI

The “maftools” package was used to evaluate mutated genes in the various risk categories, and the oncoplots display the 20 genes with the greatest mutation frequency in each group independently. The association between TMB and IMRGs-associated prognostic genotyping was analysed using the “limma” and “ggpubr” packages. The ideal cutoff of the TMB for differentiation was determined using the “survival” and “survminer” packages, and the survival curves of the tumour mutation load and the combined high-risk and low-risk groups were generated. While doing so, we also compared the two high-risk and low-risk groups’ connections with MSI.

### Drug sensitivity prediction

To compare the therapeutic effects of chemotherapy and targeted medications in high-and low-risk patients, we utilized the “pRRophetic” program (27) to estimate the semi-inhibitory concentration (IC50) values for these agents.

### Statistical analysis

The Pearson test was used for the correlation analysis. Survival in each group was tested using the Log-Rank test. The Wilcoxon test was used to compare the two sets of data. Drug sensitivity analyses were performed using R version 4.1.2. Other statistical analyses were performed using R version 4.2.0. The statistical dominance threshold was set at p < 0.05.

## Results

### Acquisition of IMRGs

**Figure 1:**
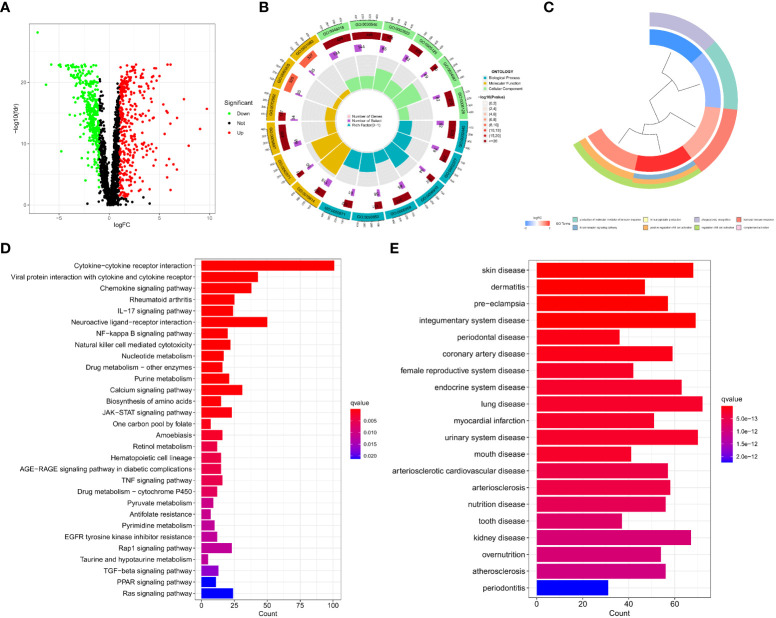
Acquisition of DEGs for IMRGs. **(A)** Volcano plot of the DEGs of IMRGs in COAD; **(B)** GO enrichment analysis; **(C)** Cluster plot analysis of GO enrichment; **(D)** KEGG analysis of the related pathways; **(E)** Results of the DO enrichment analysis.

By performing a differential analysis of 2597 IMRGs. 700 DEGs from IMRGs, with 354 up and 346 downregulated genes, were acquired and volcano for presentation (**Figure 1A**). GO enrichment analysis of these DEGs found that they were mainly involved in cellular components and molecular functions, and were less involved in biological processes (**Figure 1B**). The clustering plot of GO shows that these DEGs are mainly enriched in the production of molecular mediator of immune response, B cell receptor signaling pathway, immunoglobulin production, positive regulation of B cell activation, phagocytosis, recognition, regulation of B cell activation, humoral immune response, complement activation (**Figure 1C**). The KEGG results showed that the main enrichment in the cytokine-cytokine receptor interaction, viral protein interaction with cytokine and cytokine receptor, chemokine signaling pathway, rheumatoid arthritis, IL−17 signaling pathway, neuroactive ligand-receptor interaction, NF−kappa B signaling pathway, and natural killer cell mediated cytotoxicity (**Figure 1D**). Finally, DO disease enrichment analysis showed that diseases such as skin disease, dermatitis, pre−eclampsia, and integumentary system diseases were associated with IMRGs (**Figure 1E**).

### Identification of subtypes, TME features, and functional enrichment of IMRGs in COAD

**Figure 2:**
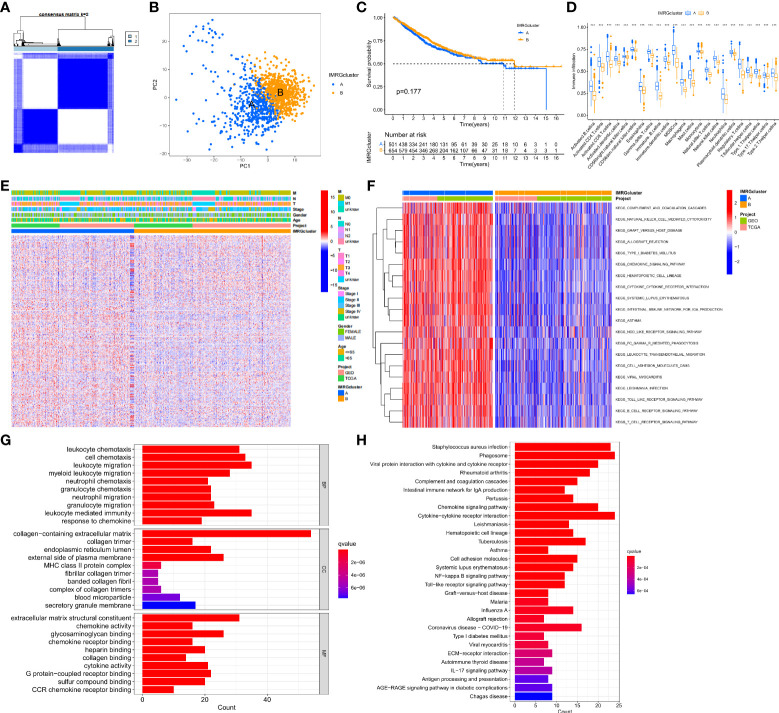
Subtype, clinicopathological, and biological characteristics of the IMRGs. **(A)** Consensus cluster analysis to construct the consensus matrix diagram of the two related regions; **(B)** PCA shows the difference between the two subtypes; **(C)** Comparative analysis of the OS rate between the two subtypes; **(D)** Differences in immune cell infiltration abundance between the two subtypes; **(E)** Heatmap of the differential clinicopathological features and IMRGs expression levels between the two different subtypes; **(F)** GSVA of the biological pathways between the two different subtypes, red: Activation pathway, blue: Inhibition pathway; **(G,H)** GO and KEGG analyses of DEGs between different IMRGs subtypes. ***p < 0.001.

In this study, the DEGs of the obtained immune and metabolism-related genes were classified by consensus clustering analysis. By adding a cluster variable (k) ranging from 2 to 9, the results found that the IMRG cluster with k = 2 is the best choice, namely, IMRG cluster A (n = 529) and IMRG cluster B (n = 682) (**Figure 2A**). The PCA analysis showed good discrimination between the two IMRG clusters (**Figure 2B**). Further mapping of the Kaplan-Meier curves of the OS of COAD patients in the two IMRG clusters showed no significant difference between the two subtype groups (p = 0.177; **Figure 2C**). Immune cell abundance showed that immune-activated cells in patients with the IMRG cluster A group were more abundant than those in patients with the IMRG cluster B group. It was shown that both groups had substantially different immune cell content for 23 of the cell proportions studied (p < 0.05; **Figure 2D**). Meanwhile, we show the relationship between two cell copper death clusters and clinicopathological features in the form of a heatmap. It mainly includes the gender (female or male), age (< = 65 or > 65 years), TNM, stage (**Figure 2E**). The results of the GSVA analysis showed that the IMRG cluster A was significantly enriched in the immune-activated pathways, such as natural killer cell mediated cytotoxicity, chemokine signaling pathway, cytokine-cytokine receptor interaction, NOD-like receptor signaling pathway, Toll-like receptor signaling pathway, B cell receptor signaling pathway, and T cell receptor signaling pathway (**Figure 2F**). However, IMRG cluster B presents immunosuppressive features. To explore the underlying biological behavior of the IMRG signatures, we identified 245 IMRG subtype-associated DEGs between the two groups. Additional research using GO and KEGG was undertaken to supplement the GSVA enrichment findings. The results showed that these DEGs were significantly enriched in matrix and immune function-related terms (**Figure 2G**). KEGG results showed significant enrichment in immune and inflammation-related pathways (**Figure 2H**). As a whole, our findings support the idea that IMRGs are an essential player in the immunomodulatory function of COAD development.

### Identification of gene subtypes based on DEGs

**Figure 3:**
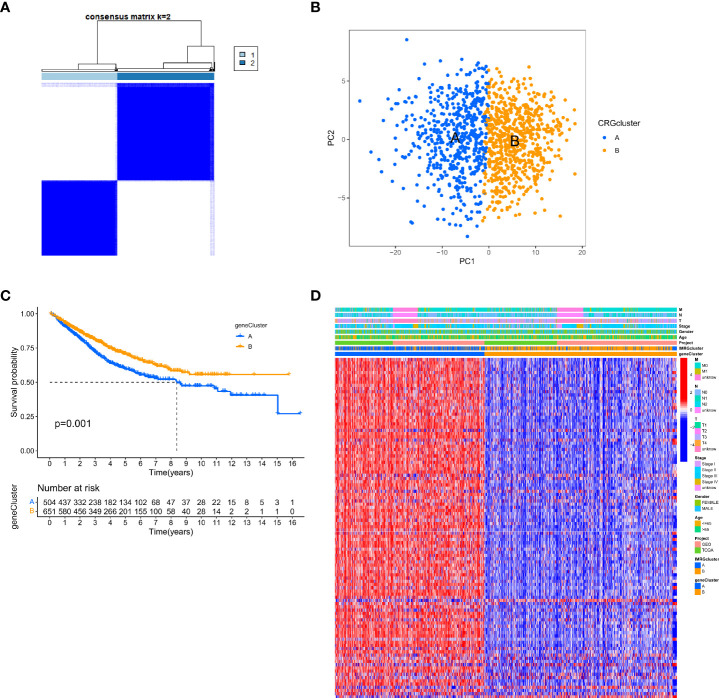
Identification of gene subtypes based on DEGs. **(A)** The consensus clustering matrix (k=2) classified the COAD patients into 2 different genomic subtypes; **(B)** PCA demonstrates variability between the two gene subtypes; **(C)** Differential analysis of OS for the 2 gene clusters; **(D)** Relationship between 2 gene clusters and clinicopathological features.

We performed univariate Cox regression analysis to estimate the prognostic value of 245 isotype-related genes and chose 106 prognosis-related genes in order to delve further into the molecular properties and prognostic value of IMRGs. At the same time, the patients were clustered according to their prognostic genes using a consensus cluster analysis. Gene cluster A (n = 529) and gene cluster B (n = 682) were identified as the two subtypes into which all samples fell after using the best cluster stability criterion (k = 2) (**Figure 3A**). The PCA showed that the two groups of genes were quite different from one another (**Figure 3B**). In comparing the two prognostic gene clusters, it was revealed that patients from cluster B had a considerably greater OS rate than those from cluster A (p < 0.001; **Figure 3C**). In the meantime, a heatmap was used to display the differences in clinicopathological aspects between the two groups (**Figure 3D**).

### Construction of a prognostic model for IMRGs

**Figure 4:**
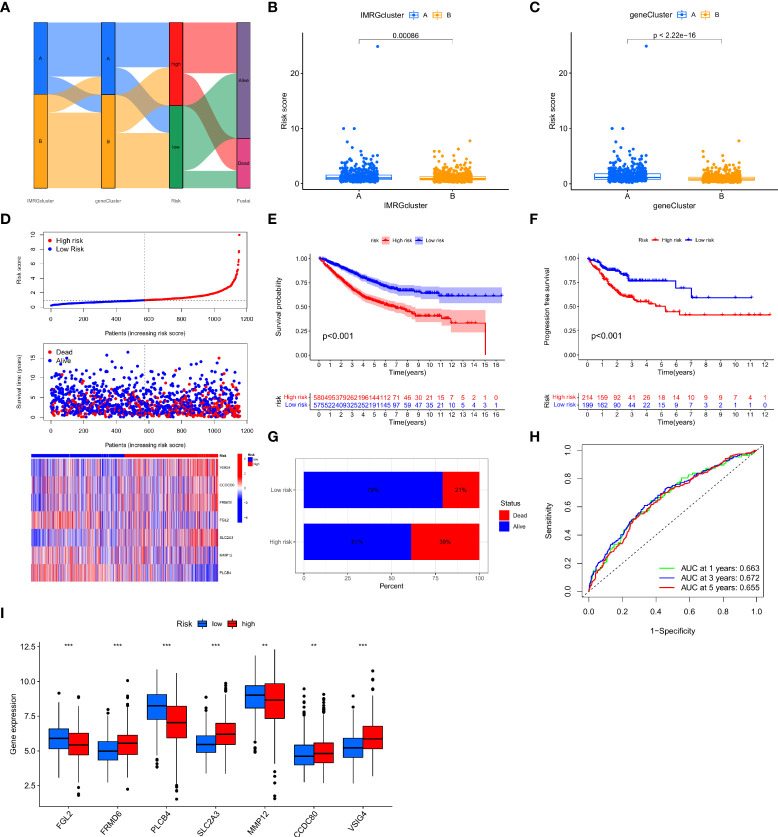
Construction and validation of the IMRG risk scores. **(A)** Sankey plots representing the relationships of IMRG clusters, gene clusters, IMRG risk scores, and survival status; **(B)** Comparison of the differential risk scores between the two IMRG clusters; **(C)** Comparison of the differential risk scores for IMRGs between the two gene clusters; **(D)** Risk distribution, survival status, and related gene expression of IMRGs risk score; **(E)** Comparison of OS rates between the high- and low-risk groups; **(F)** The PFS contrast between the high- and low-risk groups; **(G)** Survival ratio of patients in the high-risk and low-risk groups; **(H)** The ROC curves were performed according to the IMRG risk scores versus the survival sensitivity and specificity as measured at 1-, 3-, and 5-year; **(I)** Differential expression of the seven genes constructing the model in high-risk and low-risk groups; ***p < 0.001; **p < 0.01.

In this study, the IMRGs risk score was constructed based on the DEGs related to the IMRG subtype. We visualized the associations between IMRG clusters, gene clusters, IMRG risk scores, and survival status in COAD patients using a Sankey plot (**Figure 4A**). We divided the patients in a 1:1 ratio into the training group (n = 577) and the test group (n = 578). The Lasso algorithm was used for the IMRG subtype-related DEGs to obtain the coefficients for the genes selected to construct the prognostic features (**Supplementary Figure S1A** **, B**). Through the multivariate Cox regression analysis, seven genes (VSIG4, CCDC80, FRMD6, FGL2, SLC2A3, MMP12, and PLCB4) were finally determined to calculate the risk score. Among them, VSIG4, FRMD6, and SLC2A3 are the risk factors, while CCDC80, FGL2, MMP12, and PLCB4 are the protective factors. Constructing the IMRG score: Risk score = (0.4838 * expression of VSIG4) + (0.4142 * expression of FRMD6) + (0.2260 * expression of SLC2A3) + (-0.5951 * expression of CCDC80) + (-0.2569 * expression of FGL2) + (-0.2406 * expression of MMP12) + (-0.0902 * expression of PLCB4). We compared the IMRGs risk scores between two IMRG clusters and two gene clusters, and found that patients in IMRG cluster A had significantly higher risk scores than IMRG cluster B (p<0.001; **Figure 4B**). The IMRGs risk scores were significantly different between the two gene clusters, with a significantly higher risk score in gene cluster A than in gene cluster B (p < 0.001; **Figure 4C**). Patients with a lower IMRGs risk score than the median risk score were classified as low risk (n = 575), while patients with higher IMRGs risk scores were classified as high risk (n = 580). Risk score distribution plots showed that survival time decreased and deaths increased as risk scores increased. Expression of genes VSIG4, FRMD6, and SLC2A3 was positively correlated with the risk score, and genes CCDC80, FGL2, MMP12, and PLCB4 were negatively correlated with the risk score (**Figure 4D**). Meanwhile, the differential expression of these genes between patients in high and low-risk groups is shown in **Figure 4I**. The results of the risk score distribution map were confirmed in the Kaplan-Meier survival curve, where the Kaplan-Meier survival curve showed a significantly higher OS rate in the low-risk group as compared to the high-risk group (p < 0.001; **Figure 4E**). And patients in the high-risk group had a higher mortality rate (21% vs. 39%; **Figure 4G**). This indicates that the higher the risk score, the lower the OS rate, a result that is largely consistent with the OS comparison results of the gene clusters obtained from our previous analysis. Meanwhile, the analysis of progression-free survival (PFS) in the high-and low-risk groups found that the PFS in the low-risk group was significantly higher than that in the high-risk group (p < 0.001; **Figure 4F**). The impact of the IMRGs’ risk score on COAD patients’ prognoses is further supported by these findings. The AUC values of the ROC curve, including 0.663, 0.672, and 0.655, correspond to the 1-, 3-, and 5-year survival rates of the IMRG risk score (**Figure 4H**). We calculated the risk score in the training set and the test set to better test the prognostic performance of the IMRGs risk score from the internal (training set) and the external (test set), respectively. The training set and test sets were each divided into high and low-risk groups. Mortality rates were shown to be positively correlated with risk ratings in both the training and testing datasets (**Supplementary Figures S1C** **, D**). The results of the survival analysis in both the training and test sets showed better OS in the low-risk groups (p < 0.001; p = 0.004; **Supplementary Figures S1E, H**) and PFS (p < 0.001; p = 0.035; **Supplementary Figures S1F, I**). The proportion of deaths in the low-risk group was lower than in the high-risk group (19% vs. 43%; 23% vs. 35%; **Supplementary Figures S1K, L**). The predicted ROC curves at 1-, 3-, and 5-year indicate that the risk score maintains high AUC values (**Supplementary Figures S1G, J**). This demonstrates that our prediction model is accurate over both the short and long periods. Meanwhile, we performed an independent analysis of OS from the TCGA cohort and the GEO cohort. We found that the results of both the TCGA and the GEO cohort demonstrated higher OS and better prognosis for patients in the low-risk group (p < 0.001; p < 0.001; **Supplementary Figures S1M, N**).

### Clinical classification and clinical value of risk score prognostic models for IMRGs

**Figure 5:**
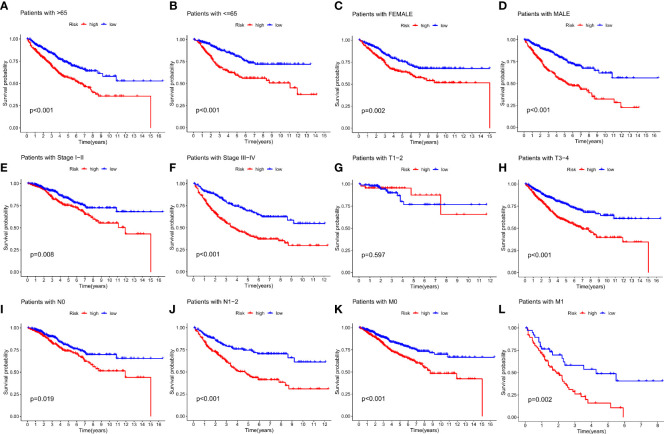
Relationship between IMRG risk scores and clinicopathologic characteristic subtypes in COAD patients. **(A, B)** age (< = 65, > 65); **(C, D)** gender (female, male); **(E, F)** stage (stageI-II, stage III-IV); **(G, H)** T (T1-2, T3-4); **(I, J)** N (N0, N1-2); **(K, L)** M (M0, M1).

To further validate the efficacy of the IMRGs risk score in its clinical prognostic application in COAD patients, we evaluated the different clinicopathological characteristics (age, gender, stage, TNM) in groups. The low-risk group had a significantly longer OS rate on age (< = 65, p < 0.001; > 65, p < 0.001; **Figures 5A** **, B**), gender (female, p = 0.002; male, p < 0.001; **Figures 5C** **, D**), stage (stage I-II, p = 0.008; stage III-IV, p < 0.001; **Figures 5E** **, F**), T (T3-4, p < 0.001; **Figure 5H**), N (N0, p = 0.019; N1-2, p < 0.001; **Figures 5I** **, J**) and M (M0, p < 0.001; M1, p = 0.002; **Figures 5K** **, L**) compared to the high-risk group. However, there was no difference in the OS rate between the two groups on T1-2 (P = 0.597, **Figure 5G**).

**Figure 6:**
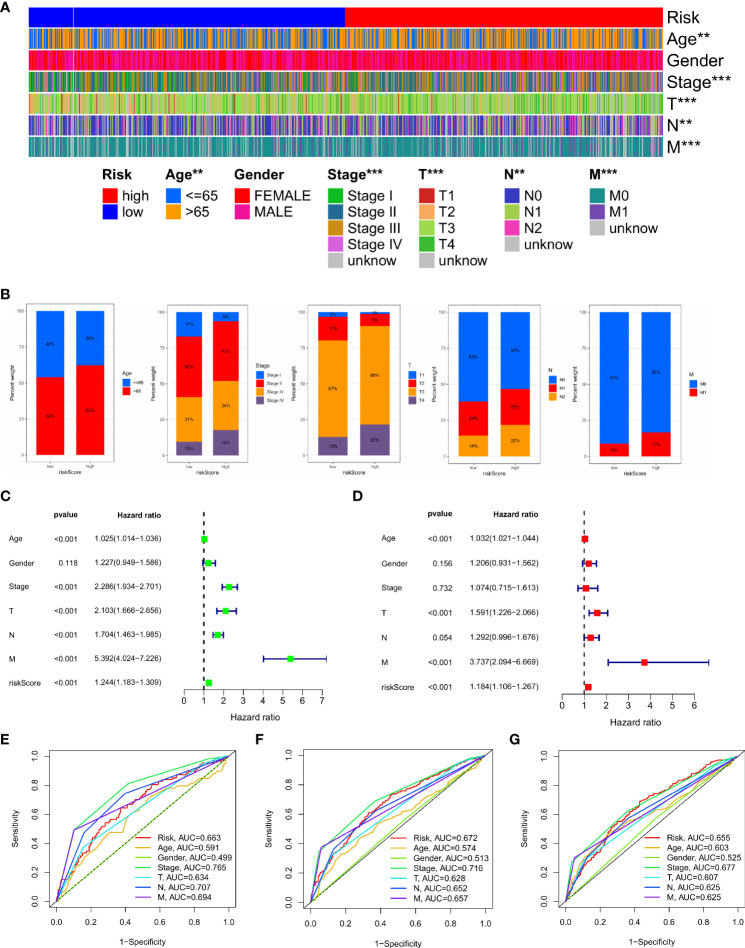
Clinical application value and independent prognostic analysis. **(A)** Heat map of the correlation between high and low risk scores and clinicopathological characteristics; **(B)** The proportion of high and low-risk scores to clinicopathological characteristics; **(C)** Univariate Cox regression analysis based on the IMRG risk score and clinicopathological characteristics; **(D)** Multivariate Cox regression analysis based on IMRG risk score and clinicopathological characteristics; **(E–G)** The ROC curve evaluates the predictive effect of the risk model at 1-, 3-, 5-year OS; ***p < 0.001; **p < 0.01.

We analysed the relationship between IMRG risk scores and a variety of clinical pathological features to learn more about the effect of IMRG risk scores on clinical characteristics. There were statistically significant variations between age, stage, and TNM and IMRG risk scores. The IMRGs risk scores were significantly higher in the age (> 65), stage IV, T4, N2, and M1 subgroups than those in the other subgroups (**Supplementary Figure S2**). Additionally, by comparing high-risk and low-risk groups for clinicopathological features, we may better understand the differences between these populations. The results showed that age, stage, T, N, and M were significantly different between the high and low-risk groups (**Figure 6A**). Their proportion in the high and low risk groups is shown in bar charts (**Figure 6B**). The results showed that patients in the low-risk group had a smaller range of clinical cancer progression than those in the high-risk group, as well as a smaller proportion of patients in the late stages of each stage. We performed both a univariate and a multivariate Cox regression analysis by combining age, gender, stage, TNM, and IMRG risk scores. Factor Cox regression analysis showed that the IMRG risk score, age, stage, T, N, and M were significantly associated with OS (**Figure 6C**). Multivariate Cox regression analysis further confirmed that IMRG risk score, age, T, and M were significantly associated with oOS, and our results demonstrated that IMRG risk score was an independent predictor of COAD prognosis (**Figure 6D**). The ROC curve combined with clinical pathological features showed that the prognostic risk model we constructed achieved good prediction accuracy at 1-year, 3-year, and 5-year OS (**Figures 6E** **–G**). Univariate/multivariate Cox regression analysis and ROC curves for clinicopathological features in the training and test sets once again validated that the IMRG risk score was an independent predictor of COAD prognosis and provided good predictive accuracy (**Supplementary Figure S3**).

### Construction and validation of the nomograms

**Figure 7:**
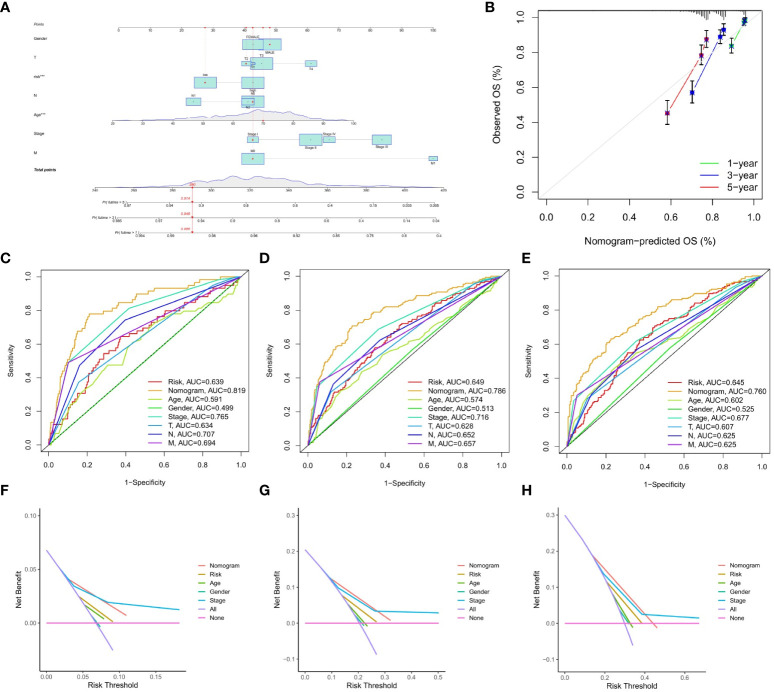
Construction and validation of the nomograms. **(A)** nomogram predicting 1-, 3-, and 5-year OS of COAD patients; **(B)** Calibration curve of the nomogram; **(C–E)** The ROC curves used to predict 1-, 3-, and 5-year of OS; **(F–H)** The DCA curves used for predicting the OS at 1-, 3-, and 5-year.

This study constructed a nomogram based on the IMRG risk score and clinicopathological factors to predict the prognosis of COAD patients, further validating the usefulness of the risk score in its clinical prognostic application in COAD patients (**Figure 7A**). Our calibration curves show that our nomogram is accurate for making 1-, 3-, and 5-year OS forecasts (**Figure 7B**). Both the risk score and the nomogram are reliable predictors, as evidenced by the ROC curves for 1-, 3-, and 5-year OS (**Figures 7C** **–E**). The DCA results demonstrated that the nomogram predicted 1-, 3-, and 5-year OS rates in COAD patients with pretty high accuracy (**Figures 7F** **–H**).

### Analysis of the immune landscape based on risk characteristics

**Figure 8:**
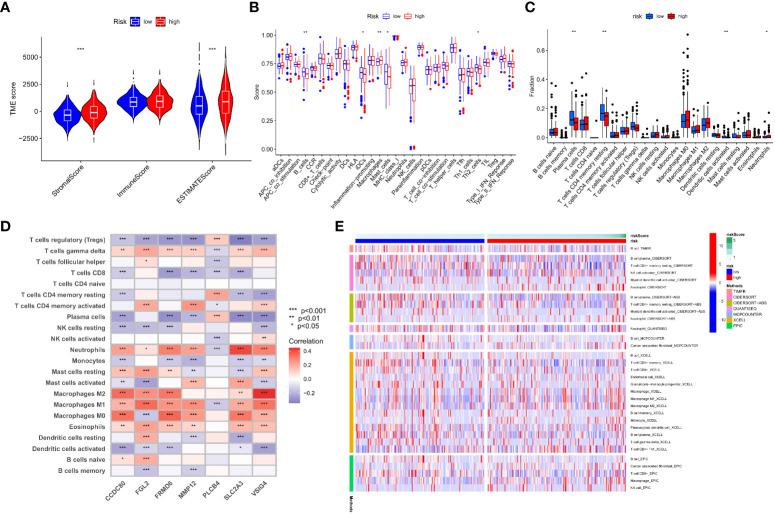
An immune landscape analysis based on risk characteristics. **(A)** The ESTIMATE algorithm evaluates the correlation of different IMRG risk score groups with TME scores; **(B)** Differential analysis of immune cells and immune function between high- and low-risk groups based on ssGSEA; **(C)** Differences in immune cell levels between high- and low-risk groups; **(D)** Correlation between immune cell abundance and the seven genes for constructing the model; **(E)** Heatmap shows the expression differences of each immune cell in the high- and low-risk groups based on different algorithms; ***p < 0.001; **p < 0.01; *p < 0.05.

TME is important in the progression and treatment of cancer, so we looked into the differences in immunological features between high- and low-risk populations. We used ESTIMATE to compare TME scores (stromalscore, immunescore, and estimatescore) across IMRG risk categories. In comparison to the low-risk group, both the stromal and estimate scores were found to be considerably higher (p < 0.001; **Figure 8A**). The low-risk group had substantially higher ssGSEA scores than the high-risk group did for B cells, iDCs, macrophages, mast cells, and the risk of Th2 cells using differential analysis (**Figure 8B**). The low-risk group had significantly higher levels of plasma cells, CD4 memory resting T cells, activated dendritic cells, and neurophils infiltration than the high-risk group (**Figure 8C**). These findings point to the possibility that immune cell infiltration and immunological activity contribute to the improved prognosis of patients in the low-risk category. Meanwhile, we also analysed the association between seven genes for developing the predictive risk model and immune cell abundance, and we observed that most immune cells were substantially connected with seven genes (**Figure 8D**). We also looked at how the IMRG risk score was connected to the immune cell subtypes and found that 13 out of 22 immune-associated cell cells had a significant relationship to the IMRG risk score (**Supplementary Figure S4**). Seven immune cells (memory B cells, M0 macrophages, M1 macrophages, M2 macrophages, activated mast cells, nutrophils, and activated NK cells) in particular were shown to have a significant positive correlation with the IMRG risk score. The IMRG risk score was inversely related to six immune cells (naive B cells, resting dendritic cells, resting NK cells, plasma cells, CD4 memory resting T cells, and Tregs). Meanwhile, we quantified the immune infiltration and function between the two groups by using the TIMER, CIBERSORT, quanTIseq, xCell, and MCP-counter and EPIC algorithms, as presented centrally by the heatmap (**Figure 8E**). In light of these findings, the IMRG risk score may have substantial clinical treatment relevance for patients with COAD by influencing the immune microenvironment infiltration.

**Figure 9:**
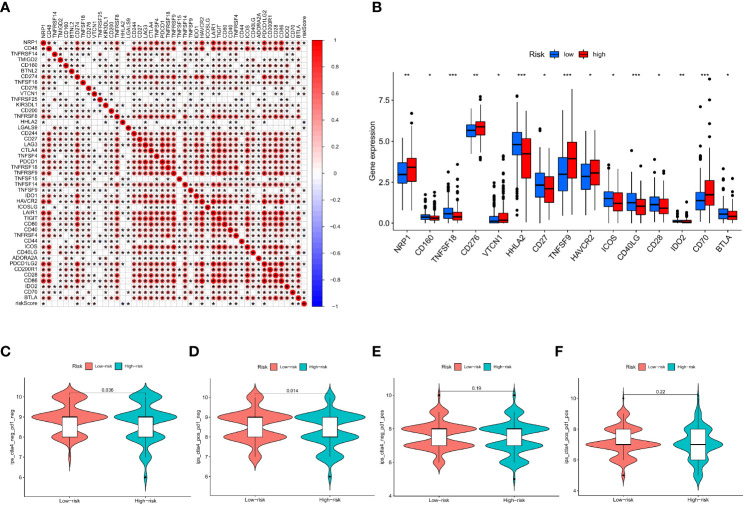
Based on the correlation between risk characteristics and immune checkpoint genes. **(A)** Correlation between the IMRG risk score and the 47 immune checkpoint genes; **(B)** Differential expression levels of immune checkpoint genes in the high- and low-risk groups; **(C–F)** The IPS evaluates the response to immunotherapy in the high- and low-risk groups; ***p < 0.001; **p < 0.01; *p < 0.05.

This research found a strong association between the IMRGs risk score and the expression of 45 immune checkpoints by assessing the connection between the IMRG risk score and 47 immune checkpoint genes (p < 0.05; **Figure 9A**). Meanwhile, there were notable differences in the expression levels of the 15 immune checkpoint genes between the high- and low-risk groups (p < 0.05; **Figure 9B**). However, differences between the two groups could not be discerned using three widely used immune checkpoint genes (CD274, CTLA-4, and PDCD1). We got the immune cell proportion score (IPS) for COAD patients from the TCIA database to learn more about how the high- and low-risk groups responds to immunotherapy. The violin plot results of the IPS score showed that the patients in the low-risk group had a better immunotherapy effect on treatment with no PD-1 and CTLA-4 inhibitors (p = 0.036; **Figure 9C**) and on treatment with CTLA-4 inhibitors alone (p = 0.014; **Figure 9D**) compared to the patients in the high-risk group. Instead of using PD-1 inhibitors alone or as a combination of CTLA-4 and PD-1 inhibitors (**Figures 9E** **, F**).

### Correlation between IMRG risk score and MSI and TMB in COAD patients

**Figure 10:**
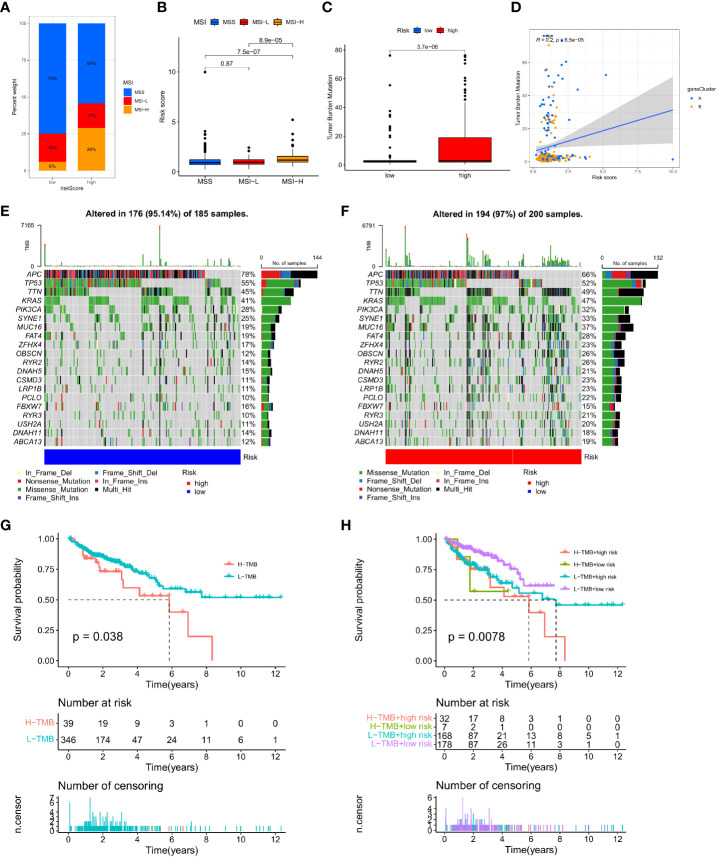
Correlation of IMRG risk score with MSI and TMB in COAD patients. **(A, B)** Relationship between the IMRG risk score and the MSI; **(C)** Comparison of TMB differences in the high- and low-risk groups; **(D)** Spearman correlation analysis of IMRG risk score and TMB; **(E, F)** Oncoplots of somatic mutations established by the IMRG risk score; **(G)** Prognostic analysis of the TMB; **(H)** Prognostic analysis between the IMRG risk score and TMB.

Changes in MSI and TMB can affect the effect of the immunotherapy that patients receive. This study found that the proportion of MSS and MSI-L in the low-risk group was higher than in the high-risk group, while the proportion of MSI-H in the high-risk group was significantly higher than in the low-risk group (29% vs. 6%; **Figure 10A**). Patients in the MSI-H group had higher risk scores than those in the MSI-L and MSS groups (p < 0.001; **Figure 10B**). Immunotherapy was especially helpful for high-risk patients. We analysed the TMB of high- and low-risk groups and found statistically significant variations between the two (p < 0.001; **Figure 10C**). Moreover, the TMB in the three gene clusters was positively correlated with the IMRG risk score (p < 0.001; **Figure 10D**). We compared the TMB conditions in the low-risk and high-risk groups, where the mutation frequencies of APC and TP53 were higher than in the high-risk group, but lower in other genes than in the high-risk group (**Figures 10E** **, F**). Considering the importance of TMB in clinical prognostic value, we did a survival prognosis study by classifying COAD patients into high-TMB and low-TMB groups according to mutation frequency. Patients with low-TMB had a greater chance of surviving, and the OS rate was higher than that of patients with high-TMB, according to the findings (p = 0.038; **Figure 10G**). Meanwhile, we performed a subgroup survival analysis based on the TMB combined with the IMRG risk score. The results showed that OS was lower in patients with higher risk and high-TMB compared to other subgroups (p=0.0078; **Figure 10H**).

### Drug sensitivity analysis

**Figure 11:**
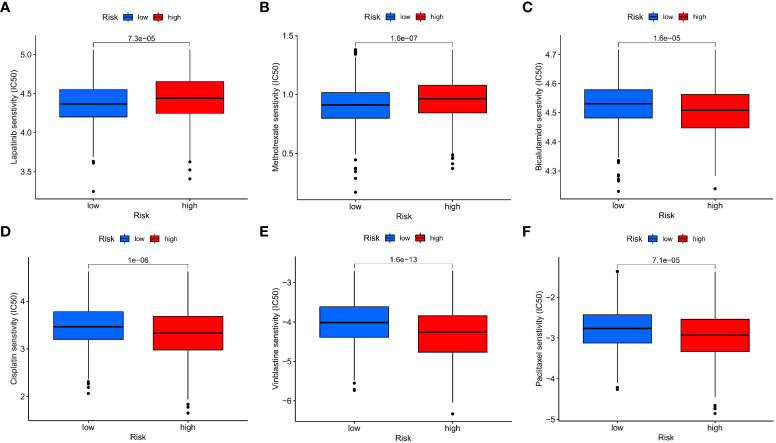
The IMRG risk score and drug sensitivity. **(A)** Lapatinib; **(B)** Methotrexate; **(C)** Bicalutamide; **(D)** Cisplatin; **(E)** Vinblastine; **(F)** Paclitaxel.

Afterwards, we looked at the connection between the IMRG risk score and the IC50 of the most commonly used chemotherapeutic and targeted medicines for COAD. We found that the IC50 values for lapatinib and methotrexate were lower among patients in the low-risk group (**Figures 11A** **, B**). But the IC50 values of other drugs, for example, bicalutamide, cisplatin, vinblastine, and paclitaxel, were lower in the patients in the high-risk group (**Figures 11C** **–F**). This result suggests that the IMRG risk score is associated with drug sensitivity.

## Discussion

The survival rate of cancer patients has increased somewhat due to the promotion of immunotherapy, but only for specific COAD patients. The complexity and heterogeneity of the immunotherapy responses observed during treatment in COAD patients may be due to the interplay between immunity and metabolism in the TME. It has been shown that immunity and metabolism are two independent key factors affecting the TME (28). And in tumor therapy, targeting inflammatory metabolic pathways can translate drug resistance into immunotherapy (29). COAD is a common cause of cancer-related death worldwide, and some studies have constructed prognostic models for immune-related genes (30). And through the metabolomics analysis of determining the serum metabolite biomarkers and related metabolic pathways in COAD (31), which helps to improve the prognostic outcomes of COAD patients. Therefore, a comprehensive comprehensive analysis of immune and metabolic gene characteristics in COAD may help us to further explore the methods and pathways to improve the prognosis of COAD patients and improve the effectiveness of immunotherapy. Therefore, a comprehensive comprehensive analysis of immune and metabolic gene characteristics in COAD may help us to further explore the methods and pathways to improve the prognosis of COAD patients and improve the effectiveness of immunotherapy. However, no relevant studies have been reported yet.

In this study, we first screened the IMRGs and then typed the extracted differentially expressed genes. With the identification of two different sets of related gene subtypes by consensus clustering, we found that IMRG cluster patients had more enriched immune cells in cluster A, and the GSVA results showed significant enrichment of IMRG cluster A on immune activation pathways. However, the KEGG analysis of DEGs of related gene subtypes showed that these genes were significantly enriched in immune and inflammation-related pathways, providing a more comprehensive complement to the biological behavior of IMRGs. To further evaluate the prognostic value of these IMRGs, we identified two gene clusters based on the DEGs between the two IMRG clusters. Our results suggest that there are significant differences in survival outcomes between the two gene clusters, and that IMRGs may serve as predictors for assessing the clinical outcomes of COAD and the response to immunotherapy. We constructed an IMRG risk scoring system and then constructed a prognostic model based on seven IMRGs (VSIG4, CCDC80, FRMD6, FGL2, SLC2A3, MMP12, and PLCB4). The accuracy of the prediction effect was evaluated and verified by the training set and the test set, and the OS rates from different cohorts (TCGA and GEO) were evaluated, respectively, and the results further verified the prognostic accuracy of our model construction. Meanwhile, we found that the IMRG risk score can affect the OS rate of patients with clinical pathological characteristic subtypes, especially when the clinicopathological factor typing of age, stage, and TNM can show good differential results, demonstrating the universality of the IMRGs-based prognostic model we constructed. Univariate and multivariate independent prognostic analyses showed that the IMRG risk score can serve as an independent predictor of COAD prognosis and has good predictive accuracy. Next, we further drew a nomogram combining the IMRG risk score and clinicopathological features and verified it. The above results indicate that IMRG prognostic features have better predictive ability in COAD patients’ survival outcomes.

V-set immunoglobulin-domain-containing 4 (VSIG4), a B7 family-related protein, is a negative regulator of T cell activation (32), which can inhibit pro-inflammatory macrophage activation by reprogramming mitochondrial metabolism (33) with pyruvate metabolism. VSIG4 expression may be associated with cancer and inflammatory diseases, and its high expression affects the poor prognosis in patients with tumors such as glioma, ovarian cancer, and gastric cancer (34) (35) (36). Studies that were similar showed that overexpressing VSIG4 in glioma U87-MG and U251-MG cells effectively reversed the apoptosis and sensitivity to temozolomide that was caused by silencing Rab18 (37). Meanwhile, the expression level of VSIG4 was also found to be significantly increased in aging tissues (e. g., adipose tissue, thymus) (38). Interestingly, in an animal model of liver damage, mice with a deletion of VSIG4 would develop severe hepatitis (39). VSIG4 has been reported to be downregulated in hepatocellular carcinoma (HCC), and its low expression is linked to a poor prognosis in patients with hepatitis B (HBV)-associated HCC (40). Coiled-coil domain-containing 80 (CCDC80) is a protein secreted by adipocytes, which is one of the adipokines that play an important role in adipocytes and systemic metabolic homeostasis (41). Related studies have shown that CCDC80 can be used as a prognostic stem biomarker to regulate the acquired drug resistance and immune infiltration in colorectal cancer (42). Currently, DRO1/CCDC80 has been identified as a tumor suppressor in the tumor microenvironment, and DRO1/CCDC80 activation in the stroma inhibits colorectal cancer growth and promotes the apoptosis of cancer cells (43). The FERM domain-containing protein 6 (FRMD6), also known as Willin, is an Ezrin/Radixin/Moesin (ERM) family protein. FRMD6 is an upstream regulator of the Hippo signaling pathway controlling tumorigenesis (44) and is responsible for coordinating mammalian peripheral neurofibroblasts (45) and antagonizing the oncogene Yes-associated protein (YAP) (46). It has now been screened and identified as a relevant factor affecting the prognosis of COAD patients (47). Fibrinogen-like protein 2 (FGL2) is involved in a variety of inflammatory and tumor signaling pathways (48). It has been identified as a novel effector molecule of Treg cells and plays an important role in regulating immune function (49). In COAD, FGL2 can be used as a new prognostic marker and an effective therapeutic target, and the overexpression of FGL2 enhances cancer cell invasion, induces epithelial mesenchymal transition (EMT), and promotes COAD invasion and metastasis (50). SLC2A3 is a glucose transporter and a central regulator of cellular energetics, and its high expression contributes to increased glucose uptake and oncogenic growth (51) (52). Related research found that overexpression of the SLC2A protein isoform is associated with poor clinical outcomes in COAD patients (53), and that SLC2A3 may participate in the immune response of COAD through PD-L1 (54). The matrix metalloproteinase (MMP) family is involved in angiogenesis, tumor invasion, and metastasis formation (55). MMP12 is expressed in a variety of tumors and can affect the tumor inflammatory response by affecting the secretion and expression of macrophages (56). Currently, it is believed that its expression function is bidirectional, that is, MMP12 expression in the tumor periphery can inhibit tumor growth while in the tumor, the expression promotes tumor growth (57) (58). At present, a study has shown that the high expression of MMP12 in the serum of COAD patients leads to the impaired overall survival of cancer patients (59). PLCB4 encodes the ß4 isoform of phosphoinositide-specific phospholipase C (PLC) isoenzymes, a superfamily orchestrating the metabolism of inositol lipids (60). PLCB4 is highly expressed in a variety of tumors and leads to a poor prognosis in cancer patients (61) (62). However, no study has specifically elucidated the mechanism of PLCB4 in the development of COAD. The association between PLCB4 expression and COAD needs further research.

The immune response in the TME is considered to be an important factor in determining tumor aggressiveness, progression, and response to immunomodulators (63). To explore the relationship of IMRGs with TIME, we analyzed the immune landscape based on immune and metabolic features. We evaluated the TME score in the high- and low-risk groups through the ESTIMATE package, while the high matrix score and the high immune score reflect the lower the purity of the tumor and the more conducive to tumor genesis and progression. In this study, the matrix score and ESTIMATE score were significantly higher in the high-risk group than in the low-risk group, which means that the IMRG risk score was positively correlated with the matrix score and ESTIMATE score and negatively correlated with tumor purity, which was unfavorable to the prognosis of cancer patients. The degree of immune infiltration significantly affects the prognosis of COAD (64). Combined with the differences in immune function scores and immune cell infiltration levels between the high- and low-risk groups. We found that the levels of T cells, B cells, mast cells, macrophages, plasma cells, and dendritic cells were all significantly higher in the patients, as seen in the IMRG low-risk group, as compared to the high-risk group. These tumor-infiltrating immune cells are the key components in regulating tumor development and treatment response (65), and play an important role in activating immune function and tumor suppression (66). Immunotherapy has been studied in a variety of solid tumors, including COAD (67), and the long-term immunotherapy-related responses and better prognosis of ICIs and MSI are significantly associated (68). The main predictive markers of treatment response in ICIs include PD-L1 expression and several biomarkers, including TMB and MSI (69). At present, although ICIs show better antitumor effects, this therapeutic intervention does not achieve the expected response in some patients (70). In particular, in COAD, only the dMMR/MSI-H tumors can achieve better clinical treatment benefits through ICI (71). In this study, we found a significant correlation between the IMRG risk score and the 45 immune checkpoint genes. However, in the high- and low-risk groups, we did not observe the expected differences in the key immune checkpoint genes CD274, CTLA-4, and PDCD1, a result that may affect the effect of immunotherapy in COAD patients. However, the IPS score showed that the CTLA-4 inhibitor alone achieved better immunotherapy in the low-risk group compared with the high-risk group. Therefore, we speculate that the IMRG risk score could facilitate the development of personalized immunotherapy strategies. Based on this study, we further investigated the correlation of the IMRG risk score with TMB and MSI. The proportion of MSI-H in the high-risk group in this study was significantly higher than that in the low-risk group, which indicates that patients in the IMRG high-risk group had a better immunotherapy benefit in COAD patients, which is basically consistent with previous reports. When we looked at the results of the analysis of the TMB group, we found that the TMB was significantly higher than that of the low-risk group. We also found that the IMRGs risk score was positively correlated with the TMB, which could be an indication of how well the high-risk group responded to immunotherapy. But combined with survival data, we found that the poor prognosis of patients with high-risk groups of mutation and immunotherapy may be the best way to solve the prognosis problem of patients with high-risk groups of mutation. Therefore, the IMRG risk score model we constructed may provide new insights for predicting immunotherapy in COAD patients. Finally, we evaluated the IC50 of different anticancer drugs in patients with high- and low-risk groups and screened potential effective treatments for high-risk groups with poor prognosis, such as bicalutamide, cisplatin, vinblastine, and paclitaxel. So, the IMRG risk score can be used as a possible predictor before chemotherapy in COAD patients, and choosing chemotherapeutic agents based on subtype helps to avoid drug resistance.

Our study demonstrates that the IMRG prognostic model constructed using a comprehensive analysis demonstrates the accuracy and clinical relevance of IMRG features by evaluation and validation of multiple datasets, including internal and external cohorts. Through immune landscape analysis and drug sensitivity screening of IMRG characteristics, our findings can help figure out the immunophenotype of COAD and design personalized immunotherapy regimens.

## Conclusion

Our exhaustive analysis of IMRG has unearthed a wide variety of regulatory mechanisms that have an effect on the TME, immunological landscape, clinicopathological characteristics, and prognosis, as well as promising medications for the treatment of the illness. It will help us learn more about the molecular processes that cause COAD and give us ideas for new ways to treat illness.

## Data availability statement

The datasets presented in this study can be found in online repositories. The names of the repository/repositories and accession number(s) can be found in the article/**Supplementary Material**.

## Ethics statement

Ethical review and approval was not required for the study on human participants in accordance with the local legislation and institutional requirements. Written informed consent for participation was not required for this study in accordance with the national legislation and the institutional requirements.

## Author contributions

Study concept and design: H-ZJ and Y-LJ. Acquisition of data: H-ZJ, BY, D-LC, F-XL, XL, ZY, and D-XT. Analysis and interpretation of data: H-ZJ. Statistical analysis: H-ZJ and Y-LJ. Drafting of the manuscript: H-ZJ and BY. Critical revision and final approval of the manuscript: H-ZJ and D-XT. Obtained funding: D-XT, ZY and BY. Study supervision: D-XT. All authors contributed to the article and approved the submitted version.

## Funding

This work was partially supported by the National Natural Science Foundation of China (No. 81860819, No. 81960818, No. 82260957, No. 82274610, No. 82160927), Guizhou Provincial Science and Technology Plan Project (Qianke-ZK (2022) 498, Qianke-ZK (2022) 487, Qianke-support (2021) 095, Qianke post-subsidy (2020) 3003, Qianke-base (2020) 1Y368, Qianke platform talent (2020) 5013), Scientific Research Innovation and Exploration Special Project in 2021 (2019YFC171250401), the National Natural Science Foundation of China in 2021 (2019YFC171250402), Guizhou Traditional Chinese Medicine Tumor Inheritance and Science and Technology Innovation Talent Base (No. Deaf leader - (2018) No. 3), the Big Health Science and Technology Cooperation Project of the First Affiliated Hospital of Guizhou University of Traditional Chinese Medicine (No. Building branch (2019) 9-2, No. Building branch (2019) 9-2-30, No. Building branch (2019) 9-2-35), Guizhou high-level innovative talent training plan (100 levels) (No. Yankehe Talents (2016) No. 4032), Yang Zhu, Guizhou Province, “Traditional Chinese Medicine Oncology” Graduate Tutor Studio (No. Teaching and research GZS- (2016) 08), TCM graduate schoolworkstation (No. Teaching and research JYSZ- (2014) 018).

## Acknowledgments

The authors would like to give their sincere appreciation to the reviewers for their helpful comments on this article and research groups for the TCGA and CEO, which provided data for this collection.

## Conflict of interest

The authors declare that the research was conducted in the absence of any commercial or financial relationships that could be construed as a potential conflict of interest.

## Publisher’s note

All claims expressed in this article are solely those of the authors and do not necessarily represent those of their affiliated organizations, or those of the publisher, the editors and the reviewers. Any product that may be evaluated in this article, or claim that may be made by its manufacturer, is not guaranteed or endorsed by the publisher.

## Supplementary material

Supplementary Figure 1 Validation of the prognostic IMRG risk score model. **(A, B)** The Lasso algorithm was used to construct the coefficients for genes with prognostic features; **(C, D)** Risk distribution, survival status, and related gene expression of IMRG risk score in the training set and test sets; **(E, H)** Comparison of the OS rate between the high-risk and low-risk groups in the training set and test sets; **(F, I)** Comparison of the progression free survival between the high-risk and low-risk groupsin the training set and test sets; **(G, J)** ROC curve of 1-, 3-, 5-year OS of IMRG risk score in the training set and test sets; **(K, L)** OS ratio of patients in the high- and low-risk groups in the training set and test sets; **(M, N)** Comparison of the OS rate between the high- and low-risk groups in the TGGA and GEO.

Supplementary Figure 2 Correlation between IMRG risk score and clinical typing. **(A)** age; **(B)** gender; **(C)** stage; **(D)** T; **(E)** N; **(F)** M.

Supplementary Figure 3 Independent prognostic analysis validation for the training and test sets. **(A, B)** The training set included univariate and multivariate Cox regression analysis based on IMRG risk score and clinicopathological characteristics; **(C–E)** The ROC curve in the training set evaluated the prediction effect of risk models at 1-, 3-, and 5-year; **(F, G)** The test set included univariate and multivariate Cox regression analysis based on IMRG risk score and clinicopathological characteristics; **(H–J)** The ROC curve in the test set evaluated the prediction effect of risk models at 1 -, 3-, and 5-year.

Supplementary Figure 4 Correlation between IMRG risk score and immune cell subtypes. **(A)** Memory B cells; **(B)** Naive B cells; **(C)** Resting dendritic cells; **(D)** M0 macrophages; **(E)** M1 macrophages; **(F)** M2 macrophages; **(G)** Activated mast cells; **(H)** Resting mast cells; **(I)** Neutrophils; **(J)** Activated NK cells; **(K)** Plasma cells; **(L)** CD4 memory resting T cells; **(M)** T cells regulatory (Tregs).
