# Supplementary material for: Development and validation of prognostic models for colon adenocarcinoma based on combined immune-and metabolism-related genes
Source: Front Oncol. 2022 Oct 31;12:1025397. doi: 10.3389/fonc.2022.1025397 (PMC9661394; doi:10.3389/fonc.2022.1025397)
Supplement: Supplementary Figure 1 — Validation of the prognostic IMRG risk score model. (A, B) The Lasso algorithm was used to construct the coefficients for genes with prognostic features; (C, D) Risk distribution, survival status, and related gene expression of IMRG risk score in the training set and test sets; (E, H) Comparison of the OS rate between the high-risk and low-risk groups in the training set and test sets; (F, I) Comparison of the progression free survival between the high-risk and low-risk groupsin the training set and test sets; (G, J) ROC curve of 1-, 3-, 5-year OS of IMRG risk score in the training set and test sets; (K, L) OS ratio of patients in the high- and low-risk groups in the training set and test sets; (M, N) Comparison of the OS rate between the high- and low-risk groups in the TGGA and GEO. [file DataSheet_1.pdf]

Supplementary Material

1 Supplementary Figures

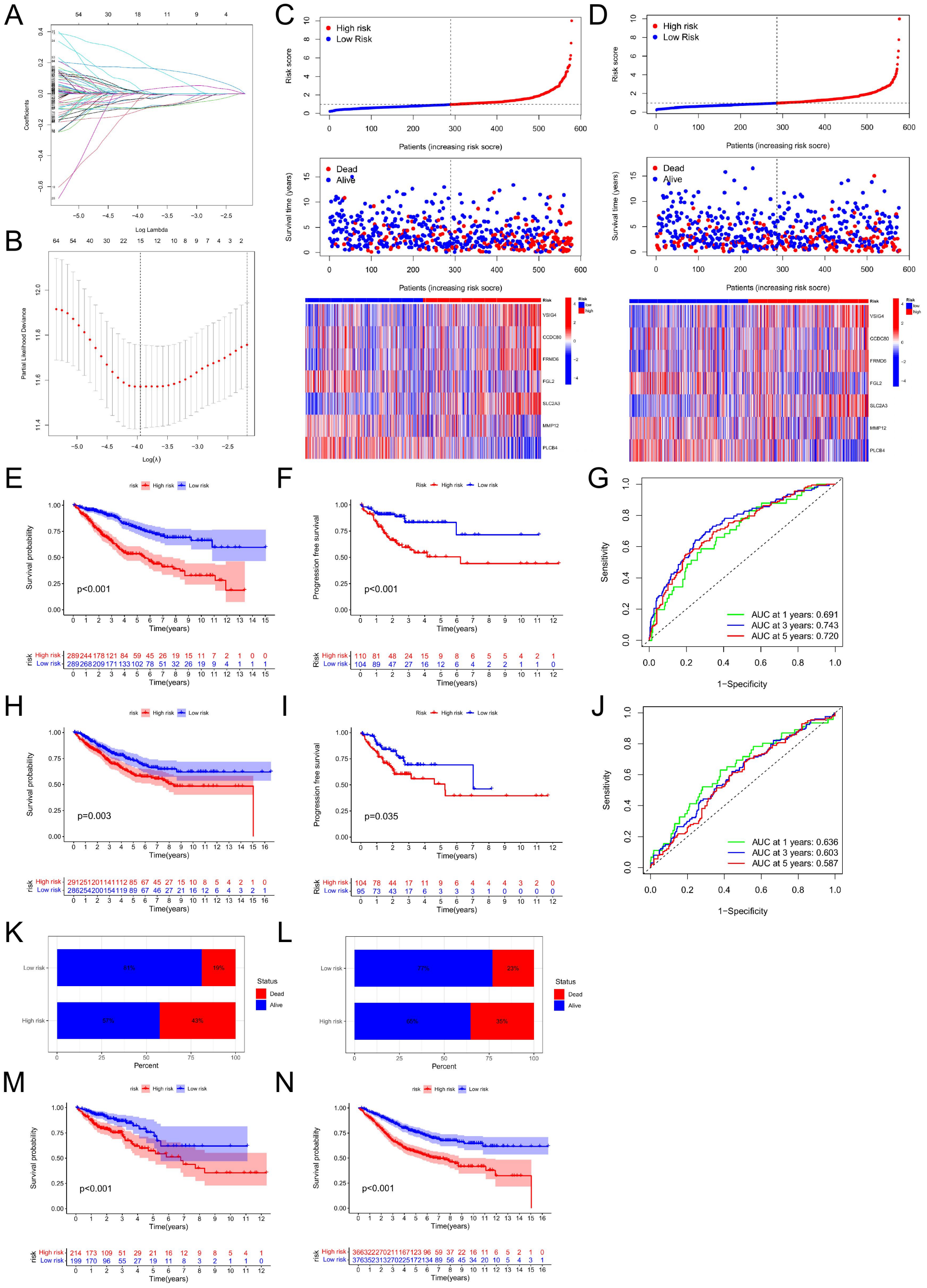

Supplementary Figure S1. Validation of the prognostic IMRG risk score model. (A-B) The Lasso algorithm was used to construct the coefficients for genes with prognostic features; (C-D) Risk distribution, survival status, and related gene expression of IMRG risk score in the training set and

test sets; (E, H) Comparison of the OS rate between the high-risk and low-risk groups in the training set and test sets; (F, I) Comparison of the progression free survival between the high-risk and low-risk groupsin the training set and test sets; (G, J) ROC curve of 1-, 3-, 5-year OS of IMRG risk score in the training set and test sets; (K-L) OS ratio of patients in the high- and low-risk groups in the training set and test sets; (M-N) Comparison of the OS rate between the high- and low-risk groups in the TGGA and GEO.

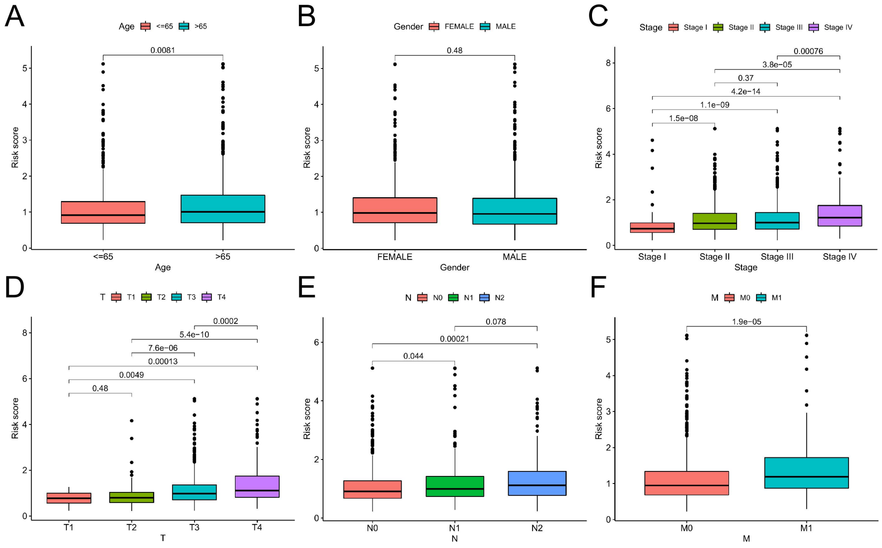

Supplementary Figure S2. Correlation between IMRG risk score and clinical typing. (A) age; (B) gender; (C) stage; (D) T; (E) N; (F) M.

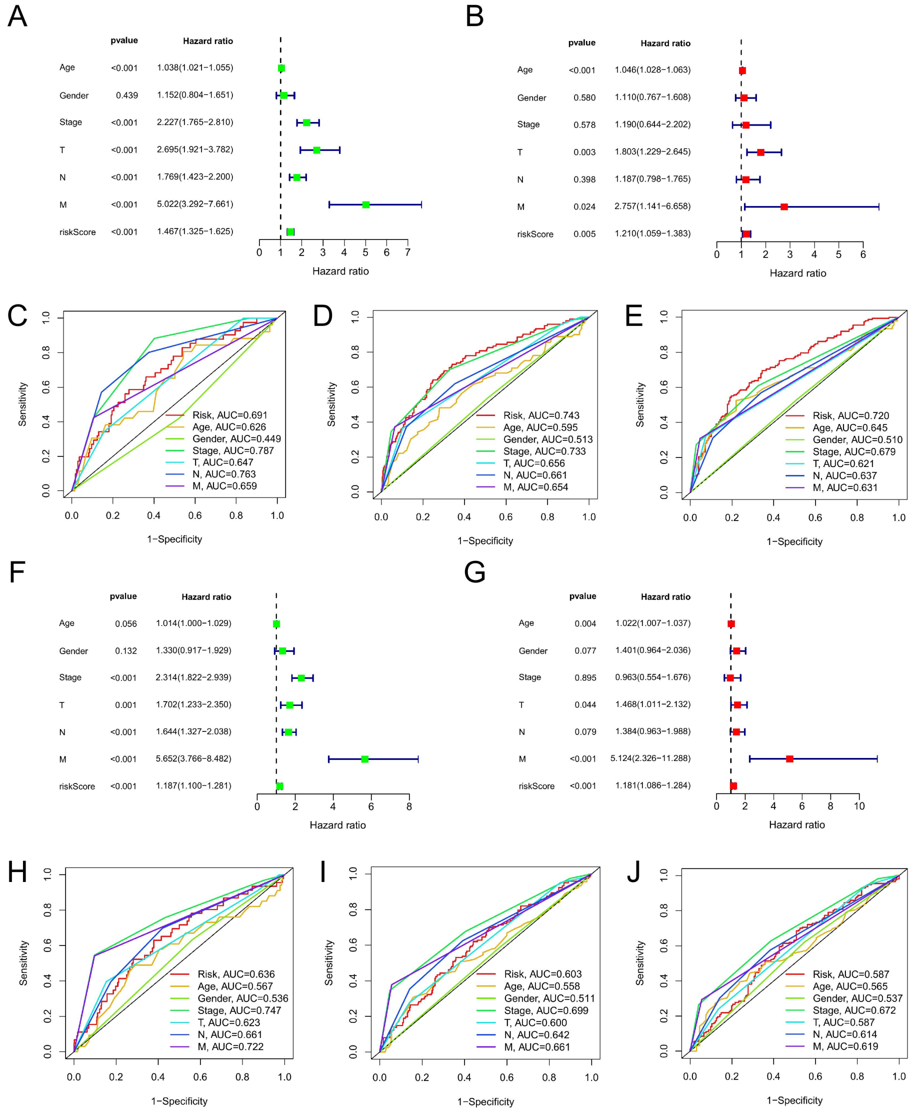

Supplementary Figure S3. Independent prognostic analysis validation for the training and test sets. (A-B) The training set included univariate and multivariate Cox regression analysis based on IMRG risk score and clinicopathological characteristics; (C-E) The ROC curve in the training set evaluated the prediction effect of risk models at 1-, 3- , and 5-year; (F-G) The test set included univariate and multivariate Cox regression analysis based on IMRG risk score and clinicopathological characteristics; (H-J) The ROC curve in the test set evaluated the prediction effect of risk models at 1 -, 3-, and 5-year.

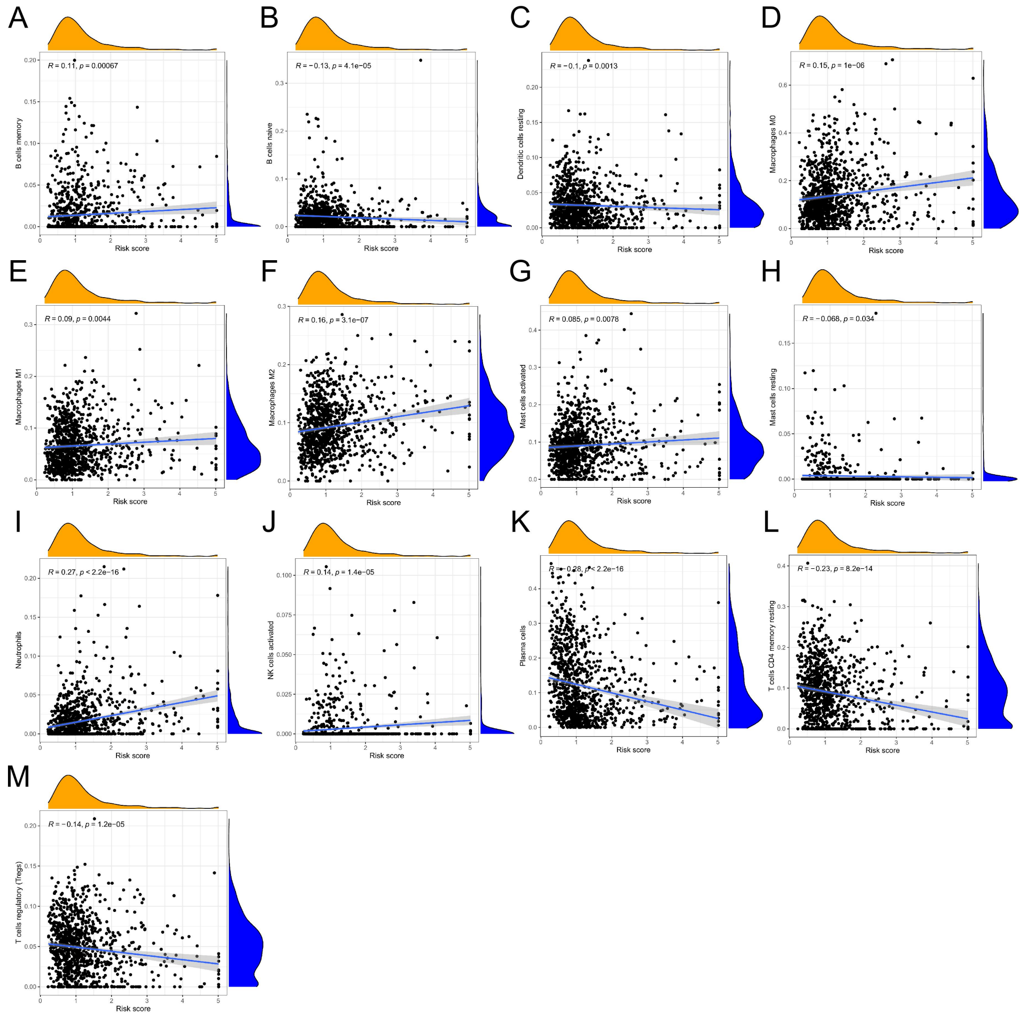

Supplementary Figure S4. Correlation between IMRG risk score and immune cell subtypes. (A) Memory B cells; (B) Naive B cells; (C) Resting dendritic cells; (D) M0 macrophages; (E) M1 macrophages; (F) M2 macrophages; (G) Activated mast cells ; (H) Resting mast cells; (I) Neutrophils; (J) Activated NK cells; (K) Plasma cells; (L) CD4 memory resting T cells; (M) T cells regulatory (Tregs).
